# The impact of clot permeability on platelet fluxes toward its surface

**DOI:** 10.1371/journal.pone.0317828

**Published:** 2025-03-25

**Authors:** Niksa Mohammadi Bagheri, Gabor Závodszky, Alfons G Hoekstra

**Affiliations:** 1 Computational Science Lab, Informatics Institute, Faculty of Science, University of Amsterdam, Amsterdam 1098 XH, The Netherlands; University of Colorado - Anschutz Medical Campus, Internal Medicine, 12700 E. 19th Ave, C272; Pulmonary, UNITED STATES OF AMERICA, Aurora, Colorado, 80045

## Abstract

Platelet aggregation is regulated by a series of chemical reactions that control platelet adhesion on a thrombogenic surface. These reactions are influenced by the complex interaction between reaction kinetics and hemodynamics. This study systematically investigates the transport of platelets, considering the interaction between flow-mediated mass transfer mechanisms and reaction kinetics as a function of clot permeability. A two-dimensional finite element model is developed to replicate static blood flow, platelet transport, and adhesion on a semi-elliptical and semi-circular structure representing permeable clots. The platelet-clot interface interactions are extensively investigated using a hindered transport model, focusing on clot permeabilities, reaction rates, and flow conditions. In the case of clots with highly reactive surfaces, an increase in clot permeability can lead up to four-fold increase in total platelet flux compared to non-permeable clots due to differences in transport environments.

## 1 Introduction

In response to vessel injury, platelets aggregate on the damaged thrombogenic surface, creating a permeable structure that facilitates the coagulation cascade and subsequent fibrin generation [1]. Platelet aggregate formation is a multi-faceted phenomenon mainly involving interactions between red blood cells, platelets, plasma proteins, and injured areas [2]. In particular, blood agonists and flow conditions [3,4] are critical factors that dictate specific thrombus composition and formation mechanisms [5,6]. Shear-induced platelet aggregates (SIPAs) [7] are characterized as permeable clots that arise from the combination of pathologic flows and sufficient concentrations of von Willebrand factor (vWF). The vWF is pivotal in promoting the platelet deposition rate by increasing platelet adhesion [8]. Thrombi can also form in backward-facing step (BFS) geometries [9] under low-flow velocity conditions and recirculation zones, leading to high concentrations of potentially activated platelets, which experience low shear stress and long residence times [10]. These areas give rise to dense accumulations of platelets and fibrin in combination with considerably lower clot permeabilities [11]. In such scenarios, platelet binding and deposition occur due to local regions experiencing a spatially or temporally induced micro shear rate gradient [12] that reaches a certain threshold [13].

Platelets are carried toward the surface of a clot through advection and diffusion[14]. Hemodynamics can also initiate diverse reaction kinetics [15,16], resulting in distinct

thrombosis scenarios. Hence, by creating an environment that is either transport-dominated or reaction-dominated [17], particularly within the region where the clot is forming [18], a unique growth rate and pattern may emerge. Tokarev et al. [19] computed the platelet flux towards the vessel wall by incorporating Smoluchowski’s theory, accounting for parameters like near-wall shear rate, collision frequency, and collision efficiency. An earlier study [20] showed that the stochasticity of platelet adhesive interactions in relation to localized flow changes plays an essential role in specifying the dynamics of the arterial thrombus shell and thrombus growth pattern [3]. It is important to note that the dynamic and static behavior of platelets and plasma proteins at the surface of a thrombus may be affected by the varying porous structure of the growing clot, thereby either intensifying or hindering the thrombosis process [21].

Blood thrombi have been characterized as heterogeneous structures, comprising a densely packed platelet core surrounded by an outer shell of loosely bonded platelets with higher permeability and dynamic behavior [22,23]. This outer layer, with its poro-viscoelastic behavior [24], significantly impacts the collision, adhesion, and embolization patterns of platelets [25]. The presence of porous structures has the potential to significantly modify flow characteristics, which, in the context of a clot, can directly influence its stability [24]. In the process of clot retraction, platelets are brought into proximity, and much of the fluid between them is expelled. As mentioned by Leiderman et al. [26], older thrombi exhibit substantially reduced porosity compared to freshly formed clots, with the study indicating a six-fold decrease in permeability for aged clots of similar composition compared to newly formed clots. According to [27], older arterial thrombi contain more fibrin fibers and fewer intervening pores, which increases clot density and may reduce thrombus permeability. Studies have reported a wide range of clot permeabilities [11,28–35], with values varying by up to seven orders of magnitude. These variations are influenced by factors such as blood flow dynamics, clot composition, and the presence or type of anticoagulants. This raises the question of why there is significant variation in permeabilities and how this parameter influences clot initiation and formation.

In analyzing the physical aspect of platelet arterial thrombosis, Du et al. [11] designed high-shear flow experiments, investigating the growth of platelet-rich white clots. Their findings revealed that the permeability of a white clot is three orders of magnitude higher than that of a red clot. Additionally, it was shown that integrating the lower reported permeabilities by others leads to an insufficient formation of interplatelet bonds, ultimately resulting in early disintegration of the thrombus.

Kim and Ku [36] reported that the presence of pores in transverse sections of a SIPA clot suggests high permeability, where this high permeability facilitates convective transport of drugs through the inner portion of the clot. On the other hand, the hindrance caused by low clot permeabilities could introduce restrictions on availability of both platelet agonists and coagulation products [21]. Muthard and Diamond [37] examined intra-thrombus permeation under controlled pressure drops in a microfluidic setup. Their findings quantitatively estimated the contracted clot permeability and the associated transport of ADP and TXA2 within the clot. According to their findings, a reduced ADP/TXA2 transport rate may also contribute to forming a dense inner thrombus core. To better understand the critical role of platelets in forming permeable clots, it is vital to investigate the underlying reasons that impact their exposure time [38] and binding rate [39] to the sub endothelium and other platelets.

The initial stages of platelet aggregate formation involve primary adhesion, followed by platelet rolling at the clot surface, which may lead to stable adhesion if platelet activation has enough time [7]. As the thrombus grows, the platelet rolling velocity increases due to the hydrodynamic force exerted on the platelets at the thrombus surface [7]. This gradual change in platelet adhesiveness leads to only a portion of platelets firmly attaching to the growing thrombus. As a result, the growth of thrombus may be limited by the capacity of platelets to transfer to the interface and adhere when the aggregate height exceeds a critical threshold [20]. So far, most theoretical models [40–43] center on platelets as chemical entities with generalized cohesive strength and binding rate, which affect the flow domain through high viscous forces in a growing region of low permeability. This approach neglects the interdependence between the distinctive permeable nature of the clots and the local fluid dynamics, especially in the shell zones of thrombus [44], in defining the impact of binding kinetics on aggregate formation. Further exploration of the interplay between binding kinetics and transport of platelets, permeabilities of clots, and hemodynamics is needed.

A recent study by Zhussupbekov et al. [45] examined the influence of surface chemistry and shear rate on thrombosis in micro-cervices. They hypothesize that this phenomenon can be attributed to the equilibrium between platelet deposition pattern and clearance. It was stated that the presence or absence of a prominent recirculation zone is believed to be the reason behind the different effects of flow rate on clot growth in various crevices. Nonetheless, to the authors’ knowledge, no research has been conducted on how variations in clot permeability and platelet adhesion chemistry affect the spatial and temporal behavior of platelets around an expanding clot in relation to hemodynamics. This study comprehensively investigates the correlation between flow-mediated and reaction-mediated mass transfer mechanisms in dictating platelet transport and deposition patterns through a mathematical framework of hindered transport of platelets. In contrast with chemical agonists, we assume that platelets can be transported toward the clot surface and may react on the clot surface but are not able to be transported into the clot, as pore sizes are probably too small to allow inter-thrombus platelet transport. This critical distinction between platelets and other relevant agonists has yet to be addressed in detail. A systematic two-dimensional numerical benchmark model is presented for simulating blood flow, platelet mass transport, and platelet adhesion to a clot surface. The model utilizes a permeable semi-circle/ellipsoid structure to represent typical generic permeable aggregate shapes in a two-dimensional domain. The model includes a specific semi-permeable boundary condition on the clot surface that allows free flow but prevents platelets from being transported into the clot. First, the specific flow patterns that govern the transportation of platelets as a function of varying hemodynamics and clot permeability ranges are studied. Then, the relationship and significance of platelet binding rates are undertaken to investigate its impact on platelet transport behavior within a thrombus’s outer layers. Platelet flux toward the surface is studied in detail to evaluate the impact of clot permeability on platelet transfer and distribution behavior surrounding a permeable clot.

## 2 Methodology

The central focus of this study is to investigate the adhesion of platelets to a permeable boundary layer of the clot, which is influenced by platelet advection and diffusion flux as well as chemical reactions. The adhesion process is assumed to depend on the flux in the normal direction to the clot boundary layer [47,48].

**Fig 1:**
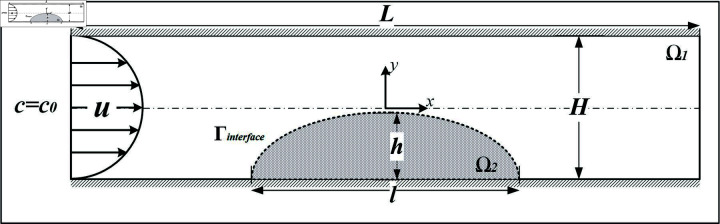
The simulation domain.

A two-dimensional flow channel of length *L* and height *H* is created (see Fig 1). A parabolic inlet velocity is assumed. At the outlet, a free flow condition is assumed. Midway the channel at the lower wall, a clot with homogeneous porosity *ε* and related permeability *κ* is situated with length *l* and height *h*. We consider a half-circle shape (*l* = *h*) and a half-elliptic shape (with *l* = 2*h*) as representations of a simplified clot shape, based on the actual thrombus shapes observed in previous studies [18,23,32,49]. A domain blockage ratio is defined as $\beta =\frac{h}{H}$. At the inlet, a homogeneous platelet concentration $c_{0}$ is assumed.

The steady-state flow of an incompressible Newtonian fluid with density *ρ* and viscosity *μ* is computed, where the effect of the porous clot domain is modeled with a body force using the Darcy-Brinkman-Forchheimer model [50]. The model was chosen for its ability to account for both viscous shear and inertial effects, offering a more accurate representation of flow dynamics around thrombi. This model surpasses the simpler Darcy law by capturing non-linear resistance under complex hemodynamic conditions. The steady-state incompressible Navier-Stokes equations are expressed in dimensionless form:


$$\begin{array}{ccc} \nabla \cdot \vec{u} & =0, & \end{array}$$ (1)

1ε2 (u→⋅∇ ⁡→)u→=−∇ ⁡(p)+1εRe ⁡ ∇ ⁡2u→−1ReDa ⁡ u→−cFDa|u→|u→, (2)

where $\vec{u}$ and *p* are the dimensionless velocity and pressure defined as $\vec{u}=\frac{{\vec{u}}^{*}}{U_{\infty }}$ and $p=\frac{p^{*}}{\rho U_{\infty }^{2}}$. The superscript * is employed to signify dimensional variables, and $U_{\infty }$ is the mean inlet velocity. In these equations, spatial coordinates are scaled by a characteristic length h, and the gradient operator $\nabla^{*}$ is transformed as $\nabla =\frac{1}{h}\nabla^{*}$. Re ⁡  and Da are the Reynolds and Darcy number, respectively, defined as $\text{Da}=\frac{\kappa }{h^{2}}$ and $\text{Re}=\frac{\rho U_{\infty }h}{\mu }\cdot C_{F}$ is the Forchheimer coefficient, $C_{F}=\frac{1.75}{\sqrt{150\varepsilon^{3}}}$. Note that in flow domain $\Omega_{1}$ (outside the clot) the terms representing this body force are zero, and the last two terms in Eq. 2 drop out (stated differently, in the free flow domain $\Omega_{1}$ one could say that Da = *∞* and *ε* = 1 resulting in the standard Navier-Stokes equations outside the clot).

The accurate relationship between the porosity (*∊*) and the permeability (*κ*) of the clot is not fully known [11]. However, it is known that within the expected range of porosities (0.3-0.7) [32], the resulting permeabilities exhibit significant changes across several orders of magnitude. Accordingly, in this study, the primary influence of clot porous structure on the flow inside the clot is investigated by dimensionless permeability (Da), following similar previous frameworks [51,52], suggesting that the main effect on the flow inside the clot is through the *Da* in the body force (Brinkman and Forchheimer drag term), with minor impact of porosity. In *Ω*2 we kept *∊* = 0 . 7 in the flow simulations while sweeping over the *Da* ranges.

The transport of the platelets is modeled via the advection-diffusion equation (*Ω*1), where we again assume a steady state, assume an isotropic and constant platelet diffusivity *D*, and express the advection-diffusion equations in dimensionless form:


$$\begin{array}{c} \vec{u}\cdot \nabla c=\frac{1}{Pe}\nabla^{2}c, \end{array}$$ (3)

where $c=\frac{c^{*}}{c_{0}}$ is the dimensionless concentration of platelets in the fluid and $c_{0}$ the platelet concentration at the inlet. The Peclet number is defined as $Pe=\frac{U_{\infty }h}{D}$. Finally, the boundary conditions for the advection-diffusion equation need to be specified. The walls of the channel are inert, which is achieved by setting the normal flux of platelets on the wall to zero, $\vec{n}\;\cdot \;\left(c\vec{u}-\frac{1}{Pe}\nabla c\right)=0$, where $\vec{n}$ is the surface normal vector. This boundary condition is also applied to the surface of the clot. Combined with an initial condition setting *c* = 0, except at the inlet, the boundary condition will act on the clot surface as a semi-permeable boundary condition, preventing platelets from entering the clot volume. However, platelets can react with the surface, where they are adsorbed, removing platelets from the solution and effectively resulting in a platelet flux towards the surface that should be included in the boundary condition. The Langmuir adsorption model is employed, where we assume that the concentration of bounded platelets on the clot surface $c_{s}$ is minimal, keeping the number of adsorption sites constant so that on the clot surface $\frac{dc_{s}}{dt}=k_{\text{ads}}c$. Applying mass balance then results in the final boundary condition on the clot surface,


$$\begin{array}{c} \vec{n}\cdot \left(c\vec{u}-\frac{1}{Pe}\nabla c\right)=\text{Dam}\cdot c \end{array}$$ (4)

with *Dam* $=\frac{k_{ads}}{U_{\infty }}$ the Damköhler number.

Simulations were performed with COMSOL 5 . 6*a*, and further details on numerical implementation and a mesh convergence study are reported in the Supporting Information.

## 3 Results

This section presents the findings of a systematic numerical analysis focusing on reactivity, hemodynamics, and permeability. The results have been evaluated for the following range of parameters: Reynolds number (1 ≤ *Re* ≤ 100 ) ; Darcy number $\left(10^{-6}\leq Da\leq 10^{-3}\right)$; Damköhler number of $\left(0\leq Dam\leq 10^{2}\right)$; and domain blockage ratio  ( *β* )  with values 0.25 and 0.5. Further details are presented in Table 1.

### 3.1 Impact of clot permeability on hemodynamics

**Fig 2:**
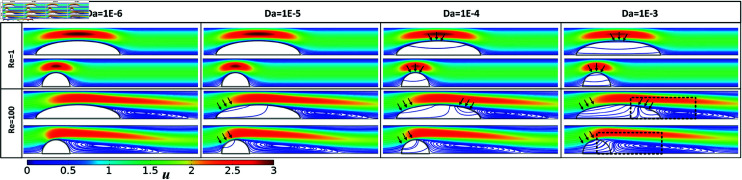
Flow streamlines and velocity magnitudes over varying ranges of Darcy numbers for semi-circular and semi-elliptical, permeable clots (*β* = 0 . 5).

**Fig 3:**
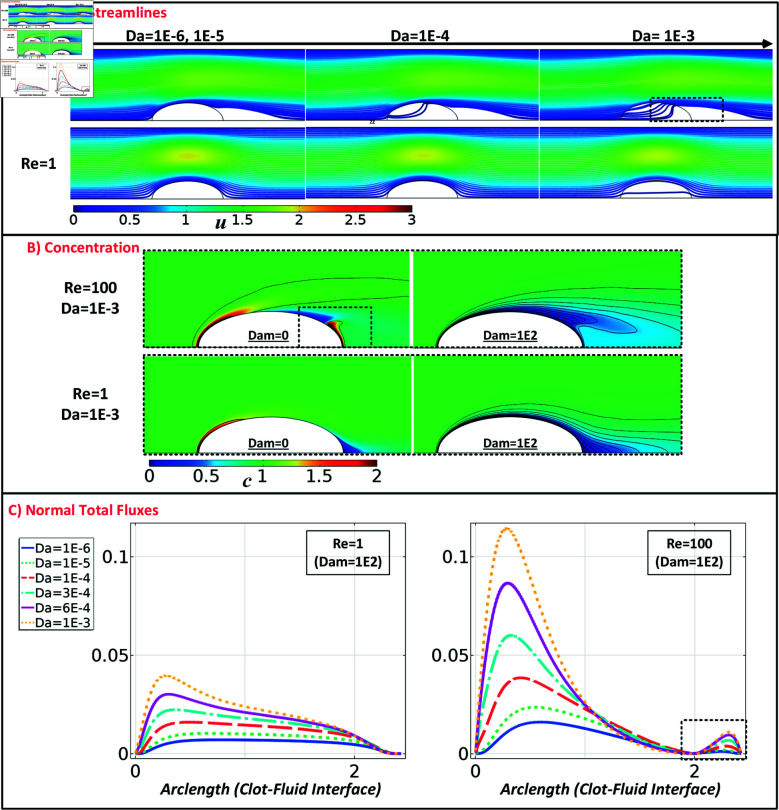
Representation of (A) flow streamlines and velocity magnitude, (B) concentration of platelets, and (C) distribution of the normal total flux to clot interface in a semi-elliptical clot with a blockage ratio of 0.25.

Flow lines and velocity magnitudes for the semi-circular and semi-elliptical permeable clots for Re = 1 , 100 , *β* = 0 . 5, and the full range of Darcy numbers is shown in Fig 2 and for *β* = 0 . 25 in Fig 3A. Results for additional *Re* numbers are shown in Fig S1.

For $Da\leq 10^{-5}$ and Re ≤ 1 the clots behave like impermeable objects. Under low-flow conditions (Re ≤  1), increasing the *Da* to $10^{-3}$ results in partial flow through the center of the clot. In contrast, under high-flow conditions *Re* > 50, a distinct alteration in flow pattern becomes apparent. For $Da\leq 10^{-6}$ clots still behave as solid objects, but already for $Da=10^{-5}$ blood flow initially moves through the upper layer of the clot (highlighted by black arrows in Fig 2). For $Da=10^{-4}$, blood flow from both sides causes an impact on the semi-elliptical shape, whereas, for the circular shape, it solely affects the upstream side of the clot. For $\text{Da}\geq 10^{-4}$ the recirculating zone starts to separate from the clot shape and shifts downstream for the semi-circular clot, while for the semi-elliptical clot, the recirculating zone moves upstream and into the permeable clot. By increasing the permeability of the clot, similar flow characteristics can be achieved at higher clot heights under comparable flow conditions. For instance, a semi-elliptical configuration with a *β* value of 0.25 induces a flow streamline similar to one with a *β* value of 0.5 with a Darcy number by one magnitude unit higher (as shown in Fig 3A). Moreover, this observation emphasizes the reduced impact of lower clot permeability on the flow behavior surrounding permeable clots in lower vessel blockage ratios. With the *β* value of 0.25, the solid behavior of a clot with a Darcy number of $10^{-5}$ is sustained at a Reynolds number of up to 100.

**Table 1:** Parameter used in simulations.

Notation	Definition	Value	Reference
Re ⁡	Reynolds number	1 to 100	*Re* values range from 0.01 [18] in small arteries to 334 for white clots growth in stenotic regions [36].
*Da*	Darcy number	$10^{-6}$ to $10^{-3}$	A wide range of reported clot permeabilities [34] $\;(2.6\pm 1.2)\times 10^{0}\left(\mu m^{2}\right)$ to $(1.5\pm 0.3)\times 10^{-5}\left(\mu m^{2}\right)$ height of the domains (30 *μm* to 3*mm* ) and varying blockage ratios.
*Dam*	Damköhler number	0 to $10^{2}$	An enhanced adhesion rate is defined as [46]: $k_{i}(x)=k_{ads}$ ⋅ $\left(1+\lambda \cdot {\dot{\gamma }}_{w}(x)\right)$ where ${\dot{\gamma }}_{w}(x)$ is the local wall shear rate, *λ* is the shear enhancement factor, $k_{ads}$ of $\left[10^{-3},10^{-4},10^{-5}\right]{ms}^{-1}$ [39]. Varying flow inlet velocities: stenotic conditions, capillary tubes, etc.
*P* *e*	Peclet number	$10^{5}$	A constant value of domain Peclet number is used (platelet diffusivity (*D*) is $1.57\;\times \;10^{-5}{mm}^{2}s^{-1}$ [40]), the Peclet-Damköhler interplay is reported by [17].
$U_{\infty }$	Average inlet velocity	1	-
$c_{0}$	Inlet concentration	1	-
*h*	Clot height	1	Dimensionless experiment set-up.
*H*	Domain height	$\frac{h}{\beta }$	Dimensionless experiment set-up with *β* = 0 . 25 , 0 . 5
*L*	Domain length	16*h*	Dimensionless experiment set-up.
-	Boundary element size	0 . 01*h*	-

In Fig 3B, two limiting scenarios are selected to show how platelet concentrations vary in a semi-elliptical clot. At a *Dam* value of 0, circulating platelets surrounding the clot interface do not react, and their concentration pattern is defined only through the processes of advection and diffusion. As a result, platelets accumulate on the upstream side of the clot due to the positive normal velocity (where a positive velocity means flow toward the surface) along the clot interface. In comparison, the negative normal velocity at the downstream side causes platelets to be washed away except in the area with overlapping wake zones. Specifically, this phenomenon leads to a positive accumulation of platelets and positive normal total fluxes behind permeable semi-elliptical clots (as shown with dashed lines, Fig 3B). In the second scenario, increased platelet reactivity accelerates the free-flowing platelets’ bonding process to the boundary layer. This substantially reduces the availability of platelets surrounding the clot boundary layer, creating a concentration gradient near the clot (see Figs. 3B and S4). Figure 3C shows the total platelet flux on the clot surface for the semi-elliptical case with a blockage ratio $\beta =0.25,\text{Dam}=10^{2}$, and *Re* =  1 and 100. An increase in clot permeability leads to a proportionate increase in total platelet flux under both low and high-flow conditions, with the largely unaffected normal total flux pattern over the clot interface, especially for low-flow conditions (see Supporting Information for other parameter settings, leading to comparable observations). This suggests higher clot growth rates for more significant clot permeability, while the shape of the growing clot is not very much influenced by the permeability. Increasing *Da* from $10^{-6}$ to $10^{-3}$ for both *Re* = 1 and 100 resulted in an approximately four-fold increase in the maximum total platelet flux. At a Reynolds number of 100, the graph depicts an abrupt rise in overall flux over the first half of the clot for high permeabilities, followed by a notable decrease in the second half. The phenomenon can increase the growth rate under high-flow conditions (Fig 3C) by amplifying the adhesion of platelets at the upstream side of permeable clots.

### 3.2 Impact of clot permeability and binding kinetics on platelet fluxes at the clot surface

**Fig 4:**
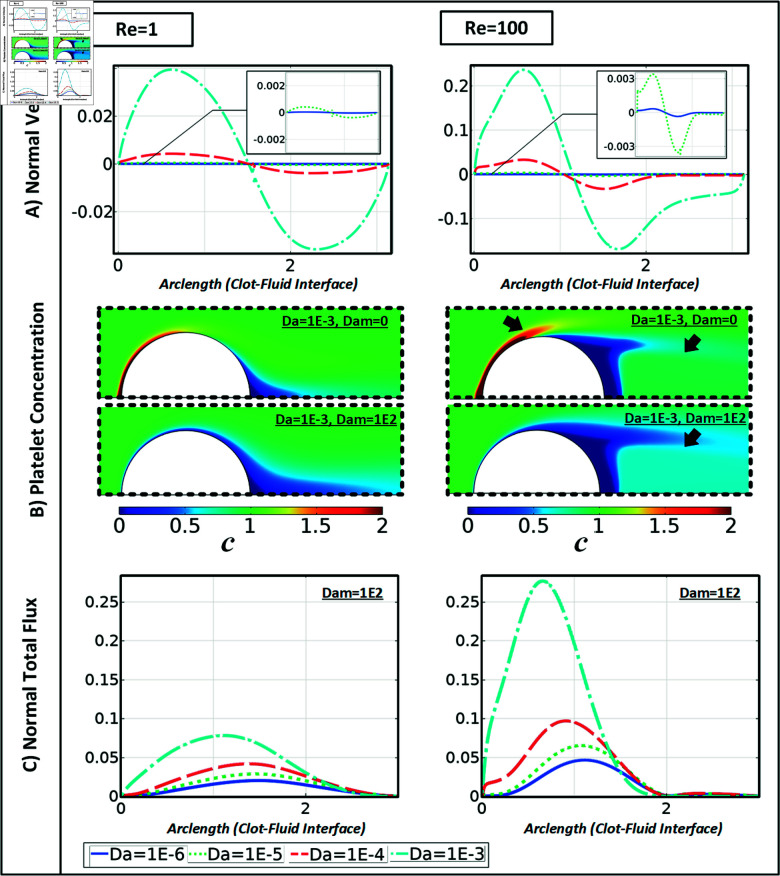
(A) Normal velocity distribution across the clot interface for a varying range of Darcy numbers, and (B) concentration of platelets, and (C) distribution of the normal total flux to clot interface in a semi-circular clot with a blockage ratio of 0.5.

Figure 4A shows normal flow velocities over the clot surface for semi-circular clots (The horizontal axis represents the arc length of the clot interface) for the full range of Darcy numbers. At small *Das*, the normal velocities are very close to zero, and for $Da\geq 10^{-4}$, substantial normal flows, primarily positive at the upstream face and negative at the downstream face, can be observed. During the assessment of the normal flow characteristics, the permeable clot exhibits a symmetrical distribution of positive and negative flow velocities along the *y*-axis, with a Reynolds number of 1 and the Darcy number of $10^{-3}$ (Fig 4A). At a Reynolds number of 100, an asymmetrical velocity pattern emerges, suggesting the existence of a re-circulation region in the clot downstream (as shown in Fig 4B by the black arrow). Figure 4B shows platelet concentrations for semi-circular clots in two scenarios. In the absence of binding reactivity, the high-flow rate (large *Pe* ) leads to significantly more platelet accumulation at the upstream face of the clot (see black arrow in Fig 4B). For Re = 100, the wake structure primarily limits the availability of platelets to this region (see black arrow in Fig 4B). Mainly, for *Dam* = 0, there is an accumulation and depletion of platelets over a more extensive area surrounding the clot interface, while for Re = 1, this impact is primarily restricted to the close vicinity of the interface. Note that for the semi-elliptical cases, both blockage ratios (see S2 Fig) display a positive normal velocity at the upstream of the clot when the wake structure shifts upward for $Da\geq 10^{-4}$ leading to a continuous build-up of platelets within this region. For $Dam=10^{2}$, most platelets arriving at the clot surface are removed from the fluid and bounded to the clot surface, rendering the concentration of free-flowing platelets in the fluid at the clot surface nearly zero. Note that this can lead to strong concentration gradients at the surface, hence strong normal positive diffusive fluxes towards the surface (see, e.g., Figs. 6 and S3). A normal total flux pattern similar to semi-elliptical cases is observed over varying ranges of Darcy numbers, except for the absence of normal total flux accumulation downstream of the clots (Fig 4C).

**Fig 5:**
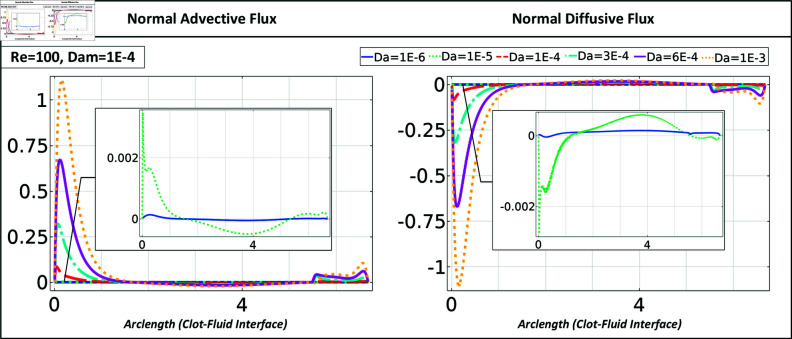
A comparison of the normal advective and normal diffusive flux at the clot-fluid interface is shown for a semi-elliptical clot with a blockage ratio of 0.5.

The total normal flux of platelets on the clot surface (The horizontal axis represents the arc length of the clot interface) consists of a normal diffusive and advective part. Figure 5 shows those fluxes for a semi-elliptical clot with a blockage ratio *β* = 0 . 5, negligible reactivity $Dam=10^{-4}$, and a range of *Da* numbers. Regardless of flow conditions, the normal diffusive flux counteracts the normal advective flux across all Darcy numbers, resulting in an almost negligible total flux. A positive normal advective flux is observed at the downstream side of the clot interface due to the overlapping of the circulating zone with the clot structure. Subsequently, this leads to a negative normal diffusive flux, promoting the accumulation of platelets in this area.

**Fig 6:**
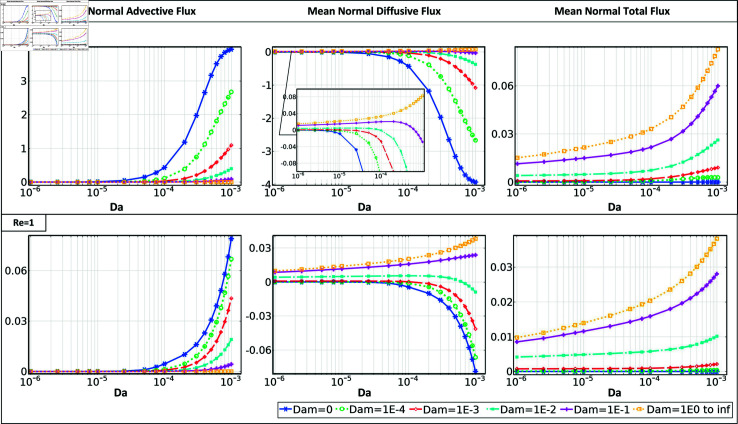
The mean normal advective and mean normal diffusive, and mean normal advective flux across the interface of a semi-circular clot with *β* = 0 . 5 for different *Da* and *Dam* ranges, at *Re* = 100 and *Re* = 1.

**Fig 7:**
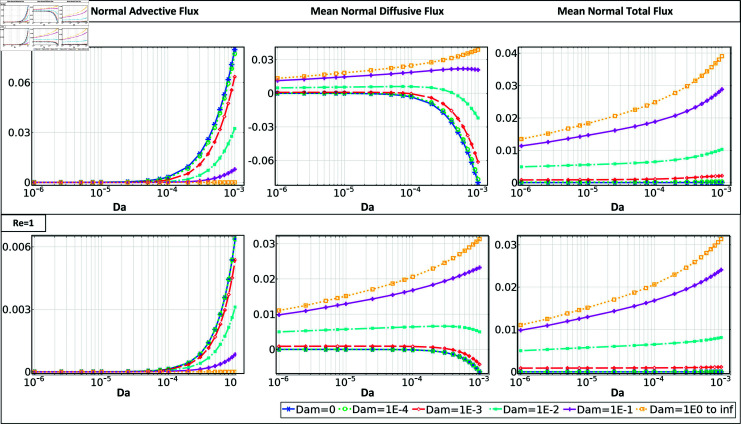
The mean normal advective, mean normal diffusive, and mean normal total flux across the interface of a semi-elliptical clot with *β* = 0 . 5 for different *Da* and *Dam* ranges, at *Re* = 100 and *Re* = 1.

Finally, mean values of normal advective, diffusive, and total flux, averaged over the clot surface, are shown in Figs. 6, 7, and S3. The mean fluxes are calculated to interpret the underlying physics, particularly in relation to clot growth behavior across varying permeability ranges. Regardless of clot permeability, *Dam* $\leq 10^{-3}$ results in a positive concentration gradient, causing the abundance of circulating platelets near the clot interface. Accordingly, both clot shapes demonstrate a balance between the normal advective and diffusive fluxes within this reactivity range, resulting in close to zero normal total flux. With Damköhler values reaching $10^{-2}$, the dynamic behavior of platelets counters a transition pattern governed by clot permeability. Except for Re ⁡  of 1 in semi-elliptical case, with such reactivity in both flow conditions, an increase of Darcy number higher than $10^{-4}$ suppresses the impact of clot reactivity and leads to platelet accumulation at the clot interface (positive gradient of concentration). The transition in platelet distribution due to variations in clot permeability can be observed, for example in low-flow conditions for a semi-elliptical clot shape *β* = 0 . 5, beginning at a Damköhler number *Dam* of $10^{-3}$ and and *Da* of $10^{-4}$. Lastly, as the *Dam* increases, a higher clot permeability is required to introduce an accumulation of platelets in the boundary layer.

During the assessment of the normal velocity arising around semi-circular clots, it was observed that the clot interface experiences a maximum normal velocity that is up to twice as high as that of semi-elliptical clots, given similar flow conditions and Darcy numbers (as depicted in Figs. 3B and S2). This phenomenon can be attributed to the higher interface curvature of the semi-circular clot shape, which causes more significant flow blockage in the vessel. In contrast, the semi-elliptical shape facilitates the partial passage of tangential flow over the interface, resulting in a decrease in normal velocity and, subsequently, a significant reduction in advective flux. This can explain why the increasing clot Darcy number, even in low-flow conditions in the semi-circular case, could cause a more predominant effect concerning clot reactivity. Regardless of clot permeability, flow condition, or shape, when the Damköhler number *Dam* reaches or exceeds $10^{-2}$, the clot interface begins to experience a reduction in circulating platelets at the reactive boundary layer. This shortage in platelet availability may start to limit further growth, as the initial flux increase transitions into a regulated, steady growth rate.

Under high-flow rate conditions, the semi-circular shape exhibits up to twice the maximum total flow and experiences up to 30 percent higher platelet total normal flux in low-flow conditions compared to semi-elliptical clots. Consistent with expectations, increasing the blockage ratio yields a greater total flux at the clot interface (see Figs. S3 and 7). The results displayed in Figs. 6 and 7 demonstrate a significant increase in the total flux, up to four times, as the clot permeability rises from a *Da* value of $10^{-6}$ to $10^{-3}$. The maximum alteration is seen in the highest reactivity condition with a *Dam* of 100.

## 4 Discussion

Research focused on computational models and experiments related to clot formation in arteries and veins has contributed valuable insights into the composition and structure of blood clots [53,54]. Notably, the timing, composition, and structure of a blood clot can be influenced by different methods of inducing thrombosis. The existence of various pathological environments underscores the importance of comprehending platelet transport characteristics for clot formation. Here, a novel computational framework is developed to analyze how influential metrics are related to platelet behavior, blood flow, and flow-mediated transport near a clot region. Particularly, the clot boundary layer experiences a specific pressure gradient due to different flow conditions and permeability, which, combined with binding kinetics, can influence platelet transport behavior. In related research, Teeraratkul et al. [55] examined how different factors, such as thrombus shape, microstructure, and degree of wall damage, impact hemodynamic characteristics and the local rotational region within the thrombus neighborhood. The study showed that leakage caused by the damaged wall can alter the pressure gradient of the boundary and significantly affect the interplay of advection and diffusion that governs flow-mediated transport. Furthermore, they highlight that the presence of small interstitial spaces in the microstructure establishes a transport regime that favors dominant diffusion and a low Peclet number. In an earlier study, Tomaiuolo et al. [18] revealed that the microenvironment within the thrombus is primarily diffusion-dominated due to the small pore size. Even when the lumen velocity was increased four times to reach 8*mm* ∕ *s*, the average clot velocity remained significantly lower than the bulk velocity. The researchers concluded that the presence and location of a tightly packed thrombus core had minimal effect on the flow velocity within the mass.

It is worth noting that previous studies have mainly focused on the characteristics outside or within clots that are tightly packed (low porosity) and under low flow conditions (Re less than 1), which reduces the impact of pressure gradient on transport characteristics. Meanwhile, at low Reynolds number and low permeability, our flow pattern result is consistent (see Fig 2) with these findings. However, recent research [36] has shown that the pores observed in the transverse clot section confirm the high permeability of the SIPA clot. The high permeability of this internal clot [11] facilitates effective drug transportation through flow movement within the clot’s domain. Our computational framework enabled the precise depiction of the diverse flow pattern observed in varying flow conditions, as well as the enhanced or hindered transport of platelets towards the permeable layer.

The visualization of flow streamlines in the Re ⁡ ≤50 and $Da\leq 10^{-3}$ regime demonstrates (see Figs. 2 and S1) that changes in clot permeability have a negligible impact on the flow profile, highlighting the essential role of diffusion in the flow-mediated transport mechanism under these circumstances. In the rest of the conditions, the clot mainly permits partial fluid flow, limiting the fluid streamlines’ reach to the attachment region of the clots. Regarding species transport in this regime, the core part of the clot remains unaffected by the advection of agonists. In agreement with earlier studies [18,55], our results indicate that as the Reynolds number decreases, the flow structure has little influence on the inner structure of the clot, especially for low permeabilities (as shown in Figs. 2 and S1). This creates a hemodynamic condition that favors diffusion [32]. It is shown that although the Darcy number has minimal impact on the streamline at low Re ⁡  (1 or lower), there is a significant difference at high Re ⁡  (greater than 50), highlighting the role of advection in the flow-mediated transport mechanism. This can directly influence platelets’ effective concentration and distribution pattern (as shown in Fig 3) and soluble agonists inside and outside growing clots at pathological high shear rates. Furthermore, we have shown that the normal flow velocity towards the clot interface and circulating zone behind the clot structure [56] regulate the concentration profile of free-flowing platelets, which could explain both the pattern and magnitude of the total normal flux in this region (as seen in Fig 4).

Here, the importance of implementing a semipermeable membrane boundary condition, which restricts platelet movement within the clot while allowing plasma flow is highlighted. This leads to two primary mechanisms that regulate the diffusion of platelets and concentration gradients at the clot boundary layer: surface reactions and the advection of platelets to the interface. It was observed that once the Darcy number surpasses $10^{-3}$, the increased permeability permits a more significant fluid influx towards the clot, resulting in a significantly higher normal total flux. Nonetheless, the platelet availability pattern derived from the normal fluxes remained largely unaffected by the increase in the Darcy number. In particular, the first half of the clot is mainly an abundance of platelets, while the clot shape determines how the wake structure behind the clot regulates platelet transport in this region. A recent study [57] indicates that the movement of platelets surrounding thrombi is influenced by the interplay between the viscoelastic forces produced by interplatelet bonds and fluid drag. They revealed that the formation of stable occlusive thrombi is contingent upon specific combinations of model parameters, including rates and the number of bonds necessary for platelet attachment. Clotting can be initiated by different mechanisms, including contact-induced activation, platelet activation due to shear rate, and interactions between platelets and red blood cells. Particularly in relatively slow flows within stagnation and recirculation zones, the activation by platelet–platelet and red blood cell (RBC)–platelet interactions becomes noteworthy. The study by Bouchnita et al. [58] underscores the significant role of recirculation zones in the stagnation of RBCs, influencing the spatial propagation and temporal dynamics of aneurysmal thrombus growth.

Carminita et al. [59] demonstrated that thrombus growth is influenced by the spatial distribution of platelets, exhibiting a transverse activation gradient from the injured endothelium towards the vessel’s periphery. According to the studies, it was determined that a thrombus consists of three distinct platelet sub-populations, resulting in a dual gradient in both the transversal and longitudinal axis of the thrombus. The highest concentration of fully activated platelets was observed in close proximity to the injury site. Our findings have demonstrated that the combined effect of the specific value range of clot permeability, flow rate, and clot shape can intensify the platelet flux at the upstream side of the clot. In a study by Rana et al. [60], it has been demonstrated that a specific geometry of an obstructed blood vessel, accompanied by a high local flow rate and subsequent spatial micro shear gradient, induces the formation of a spatial pattern of thrombus due to increased activity of vWF. In conclusion, this investigation examines how hemodynamics, clot permeability, and binding kinetics collectively impact the platelet’s transport [62,63] processes near a permeable boundary layer. Highlighting that the derived interplay between the pressure gradient and reaction rate in this boundary can hinder or expedite the movement of platelets within this layer. This research underscores the importance of accurately estimating the permeability of blood clots in different flow conditions in order to predict the initiation and development of thrombus.

## 5 Limitation

This benchmark only considers a two-dimensional setup. Key findings are expected to have similar applicability in a three-dimensional setting. In our analysis, the impact of shear-dependent binding kinetics caused by vWF and the influence of chemical-induced binding kinetics influenced by species, such as ADP were neglected, as well as the shear-induced margination of platelets. Incorporating platelet activation as a dynamic, shear-dependent variable would strengthen the model by accounting for activation thresholds, agonist availability, and local hemodynamic variations, providing a more accurate representation of thrombus development. Future model extensions could integrate variable activation states with spatial gradients and responses to shear changes, enhancing physiological relevance and advancing our understanding of thrombus initiation and growth across diverse vascular environments. In this model, a steady rather than pulsatile blood flow is assumed to simplify hemodynamic representation. However, in physiological conditions, blood flow is inherently pulsatile, particularly in arteries where pressure and flow fluctuate with each cardiac cycle. These pulsatile dynamics can influence platelet transport, adhesion, and clot growth in important ways. For example, periodic high-velocity peaks in pulsatile flow may enhance platelet transport toward the clot by intermittently increasing advective flux, thereby potentially boosting platelet deposition rates at the clot interface. Furthermore, variations in shear stress from pulsatile flow may affect platelet activation, as mechanisms such as von Willebrand factor-mediated adhesion are highly responsive to changes in shear rate. This periodic shear could create more complex adhesion and detachment behaviors than a steady-state model can accurately represent. Future studies could incorporate pulsatile flow components to better simulate oscillatory shear forces and their effects on platelet behavior, thereby enhancing the physiological accuracy of the model for applications in arterial thrombus formation. The impact of tangential platelet flux on the clot surface was not considered. Authors in [26,61] state that tangential platelet flux is also observed as a source of platelet cohesion and adhesion in the growing clot. The heterogeneity of the clot, e.g., in terms of a dense core and a more permeable shell, was also not considered. However, according to Tomaiuolo et al. [18], the transport mechanism at the clot shell zone is not significantly affected by the location and composition of the core. Integrating a heterogeneous clot structure may influence the observed internal flow pattern in high-flow conditions. The stationary geometry assumption might have limited validity for dynamic growth circumstances given the considerable impact of growing thrombi on flow patterns, flow rate, and platelet transport kinetics [46].

## 6 Conclusion

Our study employs a systematic numerical analysis of flow characteristics, platelet distribution, and transfer in a 2D rectangular domain featuring semi-circular and semi-elliptical clots under steady-state conditions as a function of clot permeability and binding reaction rates. The results show that permeability significantly influences flow patterns inside and in the vicinity of the clot in high-flow conditions, particularly for *Re* ≥ 50. Specifically, semi-circular clots subjected to high-flow rates (*Re*=100) experience up to twice the normal total flux of platelets compared to semi-elliptical clots. This study illustrates how distinctive blood flow velocity patterns, symmetrical and asymmetrical (given the circulating zone behind the clots), across a varying range of clot permeability, dictate platelets’ availability and mass transfer behavior over the clot interface. Moreover, under low- and high-flow conditions, total platelet flux toward the clot for high permeable clots can be up to four times that of an impermeable clot. In the following study, we will further investigate the contribution of clot permeability and binding kinetics in the platelet static and dynamic behavior during permeable clot growth.

## Supporting information

S1 Text Numerical implementation and validation. This file includes detailed descriptions of the grid convergence study, numerical implementation, and validation results [51].
