# Supplementary material for: The impact of clot permeability on platelet fluxes toward its surface
Source: PLoS One. 2025 Mar 25;20(3):e0317828. doi: 10.1371/journal.pone.0317828 (PMC11936424; doi:10.1371/journal.pone.0317828)
Supplement: S1 Text — Numerical implementation and validation. This file includes detailed descriptions of the grid convergence study, numerical implementation, and validation results [51]. [file pone.0317828.s001.pdf]

The Impact of Clot Permeability on Platelet Fluxes towards its Surface

Niksa Mohammadi Bagheri[1], Gabor Závodszky[1], Alfons G. Hoekstra[1],

1 Computational Science Lab, Informatics Institute, Faculty of Science, University of Amsterdam, Amsterdam 1098 XH, The Netherlands.
* a.g.hoekstra@uva.nl

Supporting Information

S1 Text. Numerical Implementation and Validation

The study utilized three distinct grids (G1, G2, and G3) to study grid convergence and varied the grid spacing δ near the clot boundary layer. Table S1 presents the reported values for the mean concentration of free-flowing platelets at clot interface (*Dam=0*) and wake length in the semi-circular case with a blockage ratio of 0.5. Mesh G3 is chosen for all simulations, as it exhibits less than a 1% variation in reported results. Finally, the computational domain was discretized using a fine mesh of 72,000 quad elements. Moreover, the present method predicted the flow around a porous semi-circular obstacle of Re=20. In a previous study, Yu et al. [64] examined the steady flow past a permeable circular cylinder for various Reynolds and Darcy numbers. Subsequently, the result of flow behavior, specifically the streamlines and wake geometry, was compared across two different Darcy numbers, revealing a good agreement (see Fig. S5 and Fig. **??** and Table S2).

Table S1. Mesh study, W_l wake length and C_m mean concentration of free-flowing platelets at clot interface

Re	Da	Grid	δ/h	W_l	C_m
100	10^{-3}	G1	0.02	3.8	0.9
		G2	0.014	3.8	0.82
		G3	0.01	3.8	0.78
	10^{-6}	G1	0.02	3.5	1.01
		G2	0.014	3.5	1.01
		G3	0.01	3.5	1.00
1	10^{-3}	G1	0.02		0.95
		G2	0.014	-	0.94
		G3	0.01		0.94
	10^{-6}	G1	0.02		1.00
		G2	0.014	-	1.00
		G3	0.01		1.00

Table S2. Comparison of wake length in two different Darcy numbers with a previous study for Re=20.

	Da=1E-5	Da=1E-6
Present study	0.95	0.85
Ref [64]	0.9	0.81

Fig S1. A) Flow streamlines and velocity magnitudes (Non-dimensional) over varying ranges of Reynolds and permeabilities and, B) Normal velocity at clot-fluid interface across varying ranges of Darcy number, for semi-elliptical, permeable clots of $\beta = 0.5$.

Fig S2. A) Contours of velocity in the y direction, B) Normal velocity distribution across the clot interface for a varying range of clot permeabilities, and C) Concentration of platelets is shown for an elliptical clot with a $\beta = 0.5$.

Fig S3. The findings of the mean normal advective, mean normal diffusive, and mean normal total flux across the interface of a semi-elliptical clot with 0.25 blockage ratio are presented for different *Da* and *Dam* ranges, at Re=100 and Re=1.

Fig S4. The concentration of platelets is shown for a varying range of clot permeabilities: A) $Dam = 0$, and B) $Dam = 100$.

Fig S5. Comparison of contours of streamlines for different Reynolds numbers (for two different porosities) with a previous study [64] (last columns); for Darcy number of 10^{-4}.

Fig S6. A schematic representation of the simulation domain and its corresponding grid.
